# Kiwi Forego Vision in the Guidance of Their Nocturnal Activities

**DOI:** 10.1371/journal.pone.0000198

**Published:** 2007-02-07

**Authors:** Graham R. Martin, Kerry-Jayne Wilson, J. Martin Wild, Stuart Parsons, M. Fabiana Kubke, Jeremy Corfield

**Affiliations:** 1 Centre for Ornithology, School of Biosciences, University of Birmingham, Edgbaston, United Kingdom; 2 Bio-Protection and Ecology Division, Lincoln University, Lincoln, New Zealand; 3 Department of Anatomy, Faculty of Medical and Health Sciences, University of Auckland, Auckland, New Zealand; 4 School of Biological Sciences, University of Auckland, Auckland, New Zealand; University of Alberta, Canada

## Abstract

**Background:**

In vision, there is a trade-off between sensitivity and resolution, and any eye which maximises information gain at low light levels needs to be large. This imposes exacting constraints upon vision in nocturnal flying birds. Eyes are essentially heavy, fluid-filled chambers, and in flying birds their increased size is countered by selection for both reduced body mass and the distribution of mass towards the body core. Freed from these mass constraints, it would be predicted that in flightless birds nocturnality should favour the evolution of large eyes and reliance upon visual cues for the guidance of activity.

**Methodology/Principal Findings:**

We show that in Kiwi (Apterygidae), flightlessness and nocturnality have, in fact, resulted in the opposite outcome. Kiwi show minimal reliance upon vision indicated by eye structure, visual field topography, and brain structures, and increased reliance upon tactile and olfactory information.

**Conclusions/Significance:**

This lack of reliance upon vision and increased reliance upon tactile and olfactory information in Kiwi is markedly similar to the situation in nocturnal mammals that exploit the forest floor. That Kiwi and mammals evolved to exploit these habitats quite independently provides evidence for convergent evolution in their sensory capacities that are tuned to a common set of perceptual challenges found in forest floor habitats at night and which cannot be met by the vertebrate visual system. We propose that the Kiwi visual system has undergone adaptive regressive evolution driven by the trade-off between the relatively low rate of gain of visual information that is possible at low light levels, and the metabolic costs of extracting that information.

## Introduction

Flight in birds is guided primarily by vision since, with the exception of high frequency echolocation found only in bats [1], no other sensory modality can provide spatial information at sufficient speed and resolution to guide flight [2]. Among birds, the nocturnal habit is derived from day-time active ancestors and, since in terrestrial environments natural ambient light levels are typically more than 1-million times lower than those during day-time [3], adaptations of sensory systems to cope with the sensory problems of night-time activity have long been of interest [3]–[5]. However, a set of fundamental constraints due to the quantal nature of light apply to any visual system, and these are manifest primarily in the trade-off between sensitivity and resolution, and the fact that any eye which maximises information gain at low light levels needs to be large [3]. This imposes exacting constraints upon vision in flying birds. Eyes are essentially heavy fluid-filled chambers and in flying birds their increased size is countered by selection for both reduced body mass and the distribution of mass towards the body core [6]. Freed from these mass constraints, it would be predicted that both flightlessness and nocturnality in birds should favour the evolution of large eyes and reliance upon visual cues for the guidance of activity. Indeed, among the largest eyes of flying birds are those of strictly nocturnal species such as owls (Strigiformes) and Oilbirds *Steatornis caripensis* [7], [8], and a general survey of eye size in birds has shown that the nocturnal habit has a strong effect on eye size relative to body mass [9]. Furthermore, among all terrestrial and aquatic vertebrates the eyes of the flightless Struthioniformes (Ostriches and their allies) and Sphenisciformes (Penguins) [10]–[12] are among the largest, suggesting that flightlessness removes an important constraint upon eye size in birds. Paradoxically, in the nocturnal and flightless Kiwi (Apterygidae), the eyes are exceptionally small with respect to body mass [12], rather than large as would be expected because of their nocturnal and flightless habits.

Five extant Kiwi taxa are recognised [13], [14]. They are endemic to New Zealand and are descended from a fauna that evolved in the absence of terrestrial mammals over a period of 80 million years [14]. Kiwi are nocturnal, flightless, cursorial birds that exploit forest floor habitats where they forage mainly for soil and surface-dwelling invertebrates [15]. Structural differences among Kiwi taxa are relatively minor, e.g. body mass, leg bone size and bill length [15]. Little is known about Kiwi sensory systems although olfaction can be used to detect food items [16] and their eyes are able to accommodate, showing that their optical system is functional [17].

To understand more fully the role of sensory systems in Kiwi behaviour, we investigated the following: eye size and the topography of visual fields as an indicator of the extent to which foraging is visually guided; minimum f-number as a measure of the light gathering capacity of the eye; the occurrence of sensory pits close to the nostrils and near the bill tip as an indicator of the extent to which non-visual cues may be involved in foraging. We also determined the extent of brain centres associated with visual processing, relative to those associated with other sensory systems, as an indicator of the relative importance of information processing from different sensory modalities.

## Results

Axial length and equatorial diameter of the two Kiwi eyes sampled = 7.0 mm. Overall eye shape was similar to that of diurnally active birds such as Common Starling *Sturnus vulgaris* and Rock Pigeon *Columba livia*. The eyes did not show the tubular shape associated with nocturnal activity in owls (Strigiformes) [7], [18]. Kiwi eye size is comparable to that of many birds of small body mass, but is markedly smaller than that of volant birds whose body mass is similar to that of Kiwi [9]. In addition, Kiwi eye size falls well outside the uniform scaling of eye diameter with body mass in birds based upon analysis of 104 species of flying birds (each species from a different family; body mass between 6 g and 4.9 kg) and within which other species of flightless birds (penguins and other ratites) also fit [12]. Assuming that Kiwi eye's optical structure is similar to that of other avian species, its focal length will be ≈0.6×(axial length) [19]. Thus, an estimate of the eye's minimum f-number (focal length/maximum entrance aperture diameter) based upon the diameter of the cornea (4.4 mm) gives a value of 0.95. This means that maximum image brightness in Kiwi eyes is similar to that of other nocturnal birds and higher than that of diurnal birds [8]. It is also similar to that of some nocturnally active mammals [20]. It can be concluded that Kiwi eyes show evidence of adaptation to lower light levels by virtue of their higher light gathering capacity. However, because of their small absolute size, the ability of kiwi eyes to retrieve spatial information at low light levels will be severely reduced, allowing the detection of only gross levels of detail within a nocturnal scene [3], unlike the situation in the larger eyed nocturnal flying birds, such as owls and Oilbirds *Steatornis caripensis* [7], [8].

**Figure 1:**
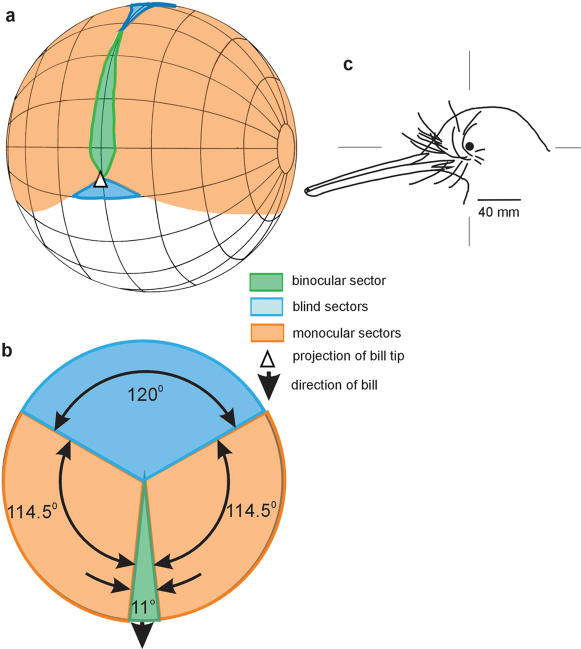
Visual fields of Kiwi. a, Perspective view of an orthographic projection of the binocular field as projected onto the surface of a sphere surrounding the bird's head. The grid shows conventional latitude and longitude at 20° intervals and the median sagittal plane of the bird's head is in the plane of the equator (which is vertical). The head is in the same posture as depicted in (c). b, Horizontal section through the visual field in the plane of maximum binocular field width which is the horizontal plane in (a) and (c). c, Drawing of a side view of a kiwi head, the bill tip projects 20° below the horizontal as shown in (a). Scale bar 40 mm.

The visual fields of Kiwi (Fig. 1) are the smallest yet reported among birds and exhibit features found in birds whose foraging is known to be guided by non-visual cues [21]. In particular, the frontal binocular field is almost non-existent. It is particularly narrow compared with those of nocturnal flying birds such as owls and Oilbirds [7], [20]. In addition, the bill falls at the very periphery of the visual field and the birds cannot see their own bill tip, This frontal visual field topography is similar to that found in birds whose foraging is guided by tactile cues from the bill rather than by vision (some dabbling ducks (Anatidae) and long-billed probing birds (Scolopacidae)) [22]. However, the total area of the binocular field is smaller and the vertical extent much less in Kiwi than in these volant tactile foragers. In these birds, the eyes are set high in the head and have monocular fields close to 180° in diameter that provide the birds with comprehensive panoramic vision about the head. In Kiwi, however, the monocular fields have a diameter of 125° and this results in a large blind area behind the head. This blind area is similar in size to that of larger eyed nocturnal birds, but in these species this results from the more forward placement of the eyes in the skull to produce a wide frontal binocular field [20]. In Kiwi, such a trade-off between wide frontal binocularity and lack of vision behind the head does not occur. Kiwi visual fields are simply small, and this, coupled with their absolutely small eye-size, indicates that the birds gather information of low spatial detail only from a very restricted area around the head. The control of forward locomotion by visual cues in birds is thought to be primarily a function of the symmetrical optical flow-fields generated in each eye within the forward facing binocular sector [21]. In Kiwi this small binocular field, coupled with low spatial resolution, clearly restricts the amount of flow-field information that is available to guide locomotion.

**Figure 2:**
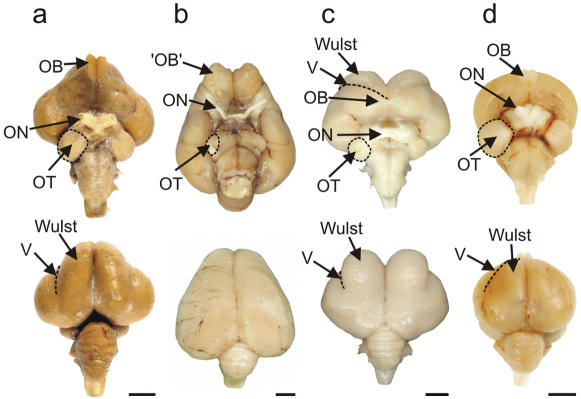
Visual processing areas of the brains of four species of birds. Ventral and dorsal views of the brains of a, Emu (diurnal, flightless); b, Kiwi (nocturnal, flightless); c, Barn Owl (nocturnal, flying), and d, Pigeon (diurnal, flying). OT: optic tectum; ON: optic nerve ; OB; olfactory bulb (which actually consists of a cortical-like sheet in the adult kiwi – see Fig. 6b); V: vallecula. Note the reduced diameter of the optic nerve in Kiwi compared with that in the three other species (see text for actual measurements). In the dorsal view of Kiwi, note the caudal extension of the large telencephalic hemispheres, which completely hide the underlying midbrain. Note also in Kiwi that there is no obvious bulge on the dorsum of the hemisphere that identifies the Wulst in species such as Barn Owl and Emu. Scale bars: Emu, 1 cm; Kiwi, Barn Owl and Pigeon: 0.5 cm.

**Figure 3:**
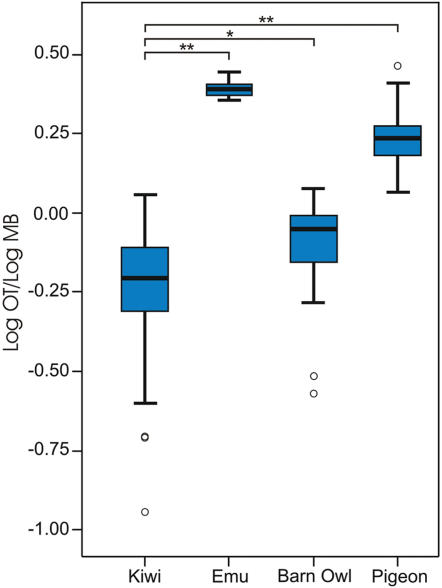
Boxplot of normalised tectal thicknesses of the four bird species. The log_10_ of the thickness of the tectum was normalised to the log_10_ of the width of the midbrain hemisection; N =  22 for kiwi, 40 for Emu, 26 for Barn Owl and 49 for Pigeon. Data was analysed using Mann-Whitney non parametric pairwise comparisons (against Kiwi). *: p≤0.005; **: p≤0.001.

**Figure 4:**
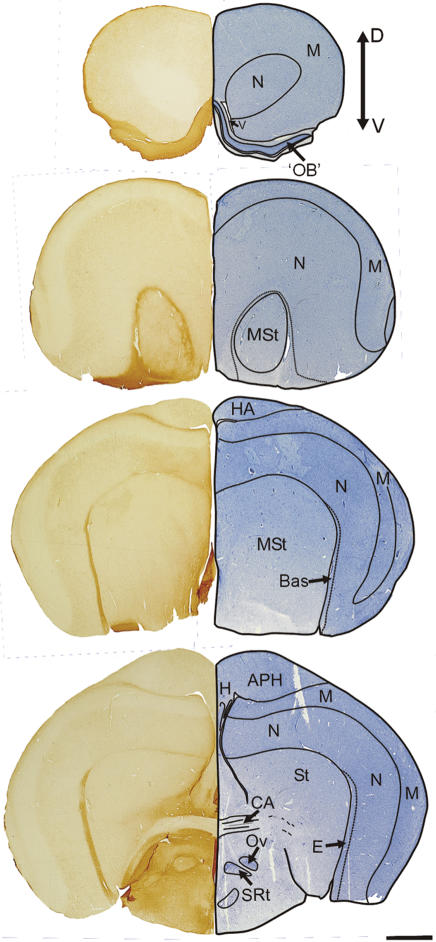
Organisation of the forebrain of the Kiwi. Series of coronal sections through the forebrain of the Kiwi (top rostral, bottom caudal). Left hemisections show the regional demarcation that results from CR-LI. APH: Parahippocampal area; Bas: Nucleus basorostralis; CA: Anterior commissure; E: Entopallium; HA: Hyperpallium apicale; M: Mesopallium; MSt; Medial Striatum; N: Nidopallium; OB: olfactory ‘bulb’; St: Striatum; H: Hippocampus; Ov: Nucleus ovoidalis; SRt: Nucleus subrotundus; v: ventricle. Scale bar: 2 mm.

In birds generally, the major retinal projection is to the highly laminated optic tectum (OT). In most birds the tectofugal pathway is by far the larger of two major visual pathways to the telencephalon, relaying in nucleus rotundus of the dorsal thalamus and terminating in the entopallium (E) embedded within the nidopallium (N). There is a smaller retinal projection to the dorsal thalamus, which then projects upon the dorsal pallium and terminates in the visual Wulst, the generally recognized homologue of the primary visual cortex of mammals. The notable exceptions to this scenario comprise those birds with more frontally placed eyes, such as owls (and some other nocturnal birds [23]), which are known to have a relatively large thalamofugal projection [24]. Craigie [25], in his examination of the Kiwi brain, commented on the reduced size, depressed form, and reduced thickness of the optic tectum, and observed reductions to all but two of its fifteen layers: the central grey and the monolaminar sixth. Here we compared the diameter of the optic nerve and thickness of the optic tectum in Kiwi (a nocturnal ratite), Emu (a diurnal ratite), Barn Owl (a nocturnal predator) and Pigeon (a diurnal, visually-guided pecking species). The results show that Kiwi have by far the smallest optic nerve diameter (Fig. 2; ON, Emu: 4.59 mm; Kiwi: 0.77 mm; Barn Owl: 1.60 mm; Pigeon: 1.58 mm), that Kiwi and Barn Owl are similar in having a relatively small optic tectum (OT), that Emu has by far the largest optic tectum and Pigeon an intermediate sized optic tectum (Figs. 2 and 3). Correspondingly, in Kiwi nucleus rotundus, the thalamic relay in the tectofugal pathway, is less conspicuous than in other birds (see also Craigie, p 298 [25]) and the entopallium (E), to which rotundus projects, appears as a narrow strip that flanks more caudal regions of the striatum (St) (Fig 4). In addition, the Wulst, the end station of the thalamofugal visual pathway, is massive in Barn Owl and Emu, moderate in Pigeon, but apparently very much reduced in Kiwi (Fig. 2). The vallecula, a groove that houses a large blood vessel and demarcates the lateral border of the Wulst in many avian species, is extremely shallow and relatively medially placed in Kiwi, with the result that the Wulst cannot be identified as a definitive bulge on the dorsum of the hemisphere, as it can in other avian species (Figs 2 and 4). Also, the Wulst does not reach the frontal pole of the telencephalon, but is displaced further caudally where it appears as the hyperpallium apicale (HA, Fig. 4). In general, these observations show a marked reduction in the size, and presumably in the visual processing capacity, of the visual centres in Kiwi, in agreement with the earlier conclusions of Craigie [25].

**Figure 5:**
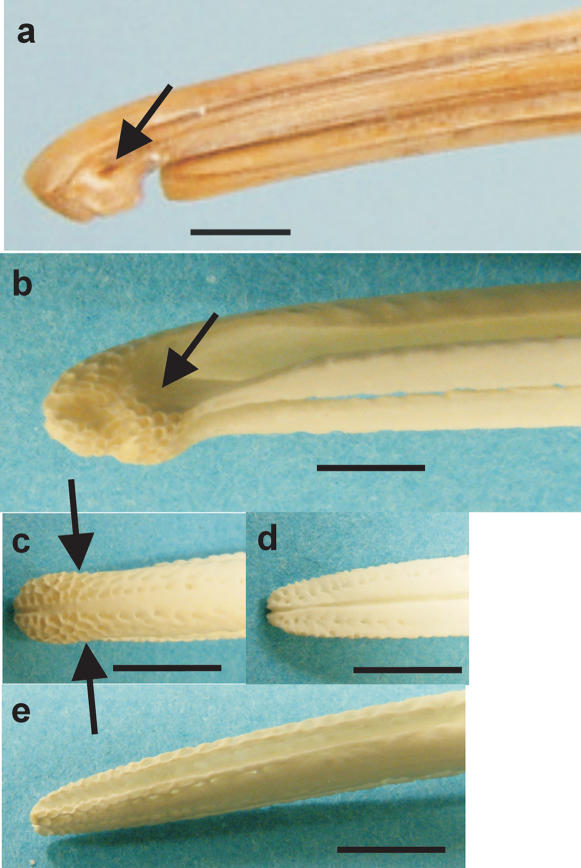
Nostrils and sensory pits at the bill tip of kiwi. a, Lateral view of the bill tip of a museum skin specimen of *A. mantelli* with the rhamphotheca intact and showing the position of the nostril (arrow). b–e, bones of the bill tip of *A. mantelli*. b latero-ventral view of the maxilla showing the complex blunt shape of the bill tip whose surface is covered with closely packed sensory pits; the approximate position of the nostril is indicated (arrow). c, dorsal view of the maxilla with the approximate positions of the nostrils indicated (arrows), d, dorsal view of the mandible showing that sensory pits are found at the bill tips within the mouth, e, latero-ventral view of the mandible showing that closely packed sensory pits cover the outer surface of the lower jaw. All scale bars 5 mm.

**Figure 6:**
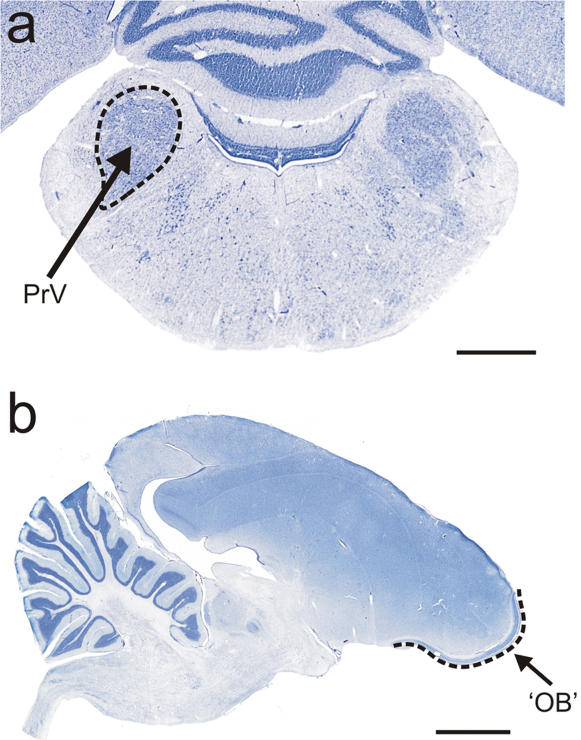
Principal sensory trigeminal nucleus and olfactory bulb. a, Cresyl Violet stained coronal section through the pons of a kiwi showing the large sensory trigeminal nucleus (PrV) that receives tactile input from the beak; b, Cresyl Violet stained sagittal section of a kiwi brain showing the olfactory bulb, which in the adult is a cortical-like sheet surrounding the frontal pole of the brain (bracketed by dashed line, and see Fig. 4). Scale bar: 5 mm.

Kiwi are unique among birds in having the opening of their nostrils close to the tip of the maxilla (Fig. 5). In all other birds, the nostrils open externally close to the base of the bill, or internally in the roof of the mouth. We provide evidence that Kiwi bill tips are the focus of both olfactory and tactile information. Inspection of prepared skulls shows that clustered around the tips of both the maxilla and mandible, on both internal and external surfaces, is a high concentration of sensory pits (Fig. 5) [26]. Such pits house clusters of mechanoreceptors (Herbst and Grandry corpuscles) protected by a soft rhamphotheca. These sensory pits function in foraging to detect objects touching or close to the bill tips [27]–[29]. In Kiwi, the sensory pits cover the entire surface of the tip of the maxilla and almost encircle the nostrils that open laterally ca. 3 mm behind the bill tip (Fig. 5), suggesting that the bill tip is a focus for gaining both tactile and olfactory information for guiding the bill when foraging. This conclusion is supported by the absolute size and histological complexity in Kiwi of the brain centres representing these modalities. For example, the principal sensory trigeminal nucleus (PrV), which receives the tactile input from the beak, is large and well-defined (cf [30]) (Fig. 6a). Furthermore, the telencephalic target of PrV, known as nucleus basorostralis (Bas), although mediolaterally narrow in Kiwi, flanks a large rostrocaudal extent of the truly massive striatum in this species (Fig. 4). Finally, the extensive olfactory cortical-like sheet that surrounds the frontal pole of the brain is the hallmark of the sensory specializations in Kiwi (Figs. 4 and 6b).

## Discussion

We have presented a range of information suggesting that although Kiwi are apparently free from weight constraints upon eye size that apply to flying birds, and that their nocturnal habits would predict a large eye size, their eyes and visual fields are in fact very small, and the visual centres serving vision are very much reduced while centres processing olfactory and tactile information are relatively large. This indicates that in Kiwi visual information is of little importance; probably a unique situation among birds. Given the relationship of Kiwi with the extinct Moa and the extant ratites, which have been noted for their large eyes [31], it seems safe to conclude that reduced reliance upon visual information is a derived characteristic in Kiwi and is probably an example of adaptive regressive evolution [32]. At some point in the evolution of Kiwi, natural selection favoured foregoing visual information in favour of other sensory information. The ecological circumstances favouring this are unclear. However, reliance upon tactile and olfactory information over visual information is found in both Kiwi and in nocturnal mammals such as rodents [33]. This suggests the independent evolution in Kiwi and in these mammals of similar sensory performance that is tuned to a common set of perceptual challenges presented by the forest floor environment at night that cannot be met by vision. Regressive evolution of visual systems have been described in both vertebrate and invertebrate animals [32], [34]. However, all of these examples have involved a complete loss of vision following colonisation of subterranean habitats devoid of light. In Kiwi, complete regression of the eye and parts of the brain associated with visual information processing has not occurred. However, while Kiwi roost and nest in burrows, their foraging habitats are not completely devoid of light [14]. Given that other flightless birds have some of the largest eyes among terrestrial vertebrates and that many flying birds of similar or smaller mass have eyes that are larger than those of Kiwi [12], it would seem that the higher cost of transport in locomotion of larger eyes is not sufficient to explain eye regression in Kiwi. We propose that regressive evolution of Kiwi vision is the result of the trade-off between the requirement for a large eye to gain information at low light levels, and the metabolic costs of extracting and processing that information [35]. It seems possible that there is an ambient light level below which the costs of maintaining a large eye and associated visual centres are not balanced by the rate at which information can be gained, and that this occurs in forest floor habitats at night.

## Materials and Methods

### Specimens

Kiwi are a group of endangered species protected under New Zealand law. We were able to work on these birds for research purposes only under strict guidelines and permits kindly issued by the New Zealand Department of Conservation and animal ethics approvals from Lincoln University

### Methods

**Visual fields** were measured in two birds (one North Island Brown Kiwi *Apteryx mantelli*; one Great Spotted Kiwi *A. haastii*). Both birds were adults and were not part of any breeding programme. To reduce disturbance to the birds, measurements were conducted on the birds' holding premises and the birds were returned to their aviaries immediately after measurements were complete. To ensure comparability of these measurement with those conducted on other birds the same procedures were used as described previously for work with a range of species (e.g. Oilbirds *Steatornis* [8], Flamingos *Phoeniconaias* [21]). Each bird was restrained with the body immobilised and the head position fixed by holding the bill. The bill was held in a specially built metal holder coated with cured silicone sealant to produce a non-slip surface. The bird's body was cradled by the bird's regular keeper/handler during the measurements. The bill holder was mounted on an adjustable mechanism and the head positioned so that the mid-point of a line joining the corneal vertices was at the approximate centre of a visual perimeter apparatus [8] that enabled the eyes to be examined ophthalmoscopically from known co-ordinates centred on the head. The perimeter's co-ordinate system followed conventional latitude and longitude with the equator aligned vertically in the birds' median sagittal plane and this co-ordinate system is used for the presentation of the visual field data (Fig. 1). Each bird's head was positioned with the plane through the eyes and bill tip pointing at an angle of approximately 20° below the horizontal. This head position approximated that which the birds adopted spontaneously when held in the hand. The projection of the bill tip when measurements were made was determined accurately from photographs and the visual field data corrected for this. The eyes were examined using an ophthalmoscope mounted on the perimeter arm. The visual projections of the limits of the frontal retinal visual field of each eye were determined as a function of elevation (10° intervals) in the median sagittal plane. To the rear of the head the limits of retinal visual field were determined at all elevations down to the horizontal. From these data (corrected for viewing from a hypothetical viewing point placed at infinity) topographical maps of the frontal visual fields and horizontal sections through the visual fields were constructed. The positions of the visual field margins in each of the birds were within 5° of each other at all elevations and the mean position of the field boundaries determined.

### Anatomy

Skins of kiwi were examined and photographed at the collections held by the Natural History Museum (Tring, UK), and skeletal materials were examined and photographed at the collections held by the Canterbury Museum (Christchurch, New Zealand). Eye size and brain structure were determined from post mortem specimens of *A. mantelli* collected in Keri Keri, New Zealand under permits issued to JRC by the New Zealand Department of Conservation (NO-16732-FAU, NO-18095-DOA). Post mortem Emu *Dromaius novaehollandiae* brains were obtained from Northland Ostrich and Emu Ltd, Kaitaia and Pigeon *Columba livia* and Barn Owl *Tyto alba* brains were obtained from specimens held at the J. M Wild lab. Kiwi (n = 2) and Emu (n = 1) brains were fixed by immersion in 4% paraformaldehyde in 0.1 M phosphate buffered saline (PBS), for 1–2 months. The brains were cryoprotected in 30% sucrose in 0.01 M PBS for 1 week and cut on a sliding microtome at 50 µm thickness in the coronal or sagittal plane. Sections were collected in PBS. Every sixth section was mounted serially onto subbed slides, stained with Cresyl Violet, dehydrated and coverslipped. Pigeon and Barn Owl brains were perfused with 4% paraformaldehyde in 0.01 M PBS, cryoprotected and cut coronally at a thickness of 35 µm and 40 µm, respectively. All tectal measurements were obtained from serial sections stained with Cresyl Violet, except for the pigeon where some measurements were taken from A Stereotaxic Atlas of the Brain of the Pigeon [36]. Measurements were obtained from 11 kiwi, 20 Emu, 14 Barn Owl, and 31 Pigeon sections. Tectal thickness was measured from the midpoint of the midbrain ventricle, orthogonally to the tectal surface. Log_10_ transformed measures were normalized to the log_10_ of the width of the midbrain at which the measurement was taken (log OT/log MB). Statistical comparisons were made using Mann Whitney U Test using SPSS v 11.

Immunocytochemistry, performed here with the sole aim of aiding the demarcation of different brain areas, was performed on Kiwi brain sections using a rabbit polyclonal antibody against calretinin (SWANT, Switzerland) at a dilution of 1∶5000. No claims as to the specificity of antibody binding are made and, therefore, we refer to the calretinin-like immunoreactivity as CR-LI. Floating sections of kiwi brains were bleached for 10 minutes in 50% methanol and 1% H_2_O_2_ to block the activity of endogenous peroxidase and washed thoroughly in 0.01 M PBS. Sections were incubated overnight at room temperature in the primary antibody in the presence of 2.5% normal serum and 0.4% Triton X-100. Sections were then incubated in an appropriate biotinylated secondary antibody (1∶300) for 1–2 hrs at room temperature, followed by streptavidin-horseradish peroxidase (1∶1000, Molecular Probes, OR) for 1–2 hrs at room temperature, and developed with a chromagen solution consisting of PBS, 0.25 mg/ml diamino benzidine tetrahydrochloride (DAB) and 0.018% H_2_O_2_. In some cases, 0.02% cobalt chloride was added to the chromagen solution to render the reaction product black. All steps in this and all other incubation procedures were separated by washes in the incubation buffer. The tissue was mounted onto subbed slides, dehydrated, and coverslipped with Permount. A brown and/or blue/black reaction product indicated positive staining for the antigen. The material was photographed on a light table using a standard photographic camera. The images were processed with Adobe PhotoShop v. 9 software to produce the final figures.
